# Supplementary material for: Population-based incidence and antimicrobial susceptibility patterns of shigellosis among children and adults from rural and urban Kenya, 2010–2019
Source: PLoS One. 2026 Mar 26;21(3):e0330888. doi: 10.1371/journal.pone.0330888 (PMC13020798; doi:10.1371/journal.pone.0330888)
Supplement: S1 Table — (DOCX) [file pone.0330888.s002.docx]

S1 Table. Characteristics of diarrhea cases who had stool sample collected compared to those who had no stool sample collected from PBIDS, Kenya, 2010-2019

|  | **Asembo** | | | **Kibera** | | |
| --- | --- | --- | --- | --- | --- | --- |
| **Characteristic** | **Stool Specimen collected** | **Stool Specimen not collected** | **P-value** | **Stool Specimen collected** | **Stool Specimen not collected** | **P-value** |
|  | n=2,017 n % | n=4,478 n % |  | n=4,074 n % | n=4,424 n % |  |
| **Age** |  |  |  |  |  |  |
| <12m | 247 (12) | 919 (21) | <0.0001 | 288 (7) | 819 (19) | <0.0001 |
| 12-23m | 207 (10) | 998 (22) |  | 442 (11) | 1167 (26) |  |
| 24-59m | 193 (10) | 665 (15) |  | 683 (17) | 1113 (25) |  |
| 5-9yrs | 87 (4) | 201 (4) |  | 417 (10) | 333 (7) |  |
| 10-17yrs | 185 (9) | 341 (8) |  | 668 (16) | 305 (7) |  |
| 18-34yrs | 389 (19) | 477 (11) |  | 905 (22) | 422 (10) |  |
| 35-49yrs | 296 (15) | 327 (7) |  | 470 (12) | 190 (4) |  |
| 50+yrs | 413 (21) | 550 (12) |  | 201 (5) | 75 (2) |  |
| **Male** | 828 (41) | 2064 (46) | 0.0002 | 1984 (48) | 2217 (50) | 0.1928 |
| **Signs/Symptoms** | | | |  | | |
| Lethargic | 38 (2) | 91 (2) | 0.6921 | 25 (1) | 37 (1) | 0.2282 |
| Unconscious | 2 (0) | 10 (0) | 0.281 | 3 (0) | 5 (0) | 0.5543 |
| Sunken eyes | 158 (8) | 245 (6) | 0.0003 | 50 (1) | 83 (2) | 0.0161 |
| Bloody stool | 446 (22) | 433 (10) | <0.0001 | 657 (16) | 206 (5) | <0.0001 |
| **Hospitalized** | 502 (25) | 732 (16) | <0.0001 | 9 (0) | 14 (0) | 0.397 |
