# Supplementary material for: Population-based incidence and antimicrobial susceptibility patterns of shigellosis among children and adults from rural and urban Kenya, 2010–2019
Source: PLoS One. 2026 Mar 26;21(3):e0330888. doi: 10.1371/journal.pone.0330888 (PMC13020798; doi:10.1371/journal.pone.0330888)
Supplement: S2 Table — (DOCX) [file pone.0330888.s003.docx]

S2 Table. Distribution of *Shigella* species over time, Asembo and Kibera in Kenya, 2010-2019

|  | **Asembo** | | | | | | | | | |
| --- | --- | --- | --- | --- | --- | --- | --- | --- | --- | --- |
|  | **2010** | **2011** | **2012** | **2013** | **2014** | **2015** | **2016** | **2017** | **2018** | **2019** |
| **Subtype** | n=35 | n=41 | n=31 | n=39 | n=83 | n=36 | n=25 | n=91 | n=40 | n=41 |
| *SHIGELLA FLEXNERI* | 20 (57) | 22 (54) | 22 (71) | 20 (51) | 51 (62) | 15 (42) | 20 (80) | 64 (70) | 25 (63) | 21 (51) |
| *SHIGELLA SONNEI* | 4 (12) | 4 (10) | 5 (16) | 1 (3) | 17 (20) | 9 (25) | 1 (4) | 13 (14) | 4 (10) | 14 (34) |
| *SHIGELLA DYSENTERIAE* | 6 (17) | 3 (7) | 3 (10) | 10 (26) | 9 (11) | 3 (8) | 3 (12) | 6 (7) | 3 (7) | 1 (3) |
| *SHIGELLA BOYDII* | 5 (14) | 11 (27) | 0 (0) | 2 (5) | 4 (5) | 6 (17) | 0 (0) | 5 (6) | 2 (5) | 5 (12) |
| *SHIGELLA SPECIES* | 0 (0) | 1 (2) | 1 (3) | 6 (15) | 2 (2) | 3 (8) | 1 (4) | 3 (3) | 6 (15) | 0 (0) |
|  | **Kibera** | | | | | | | | | |
| **Subtype** | **n=66** | **n=36** | **n=69** | **n=59** | **n=89** | **n=72** | **n=42** | **n=21** | **n=61** | **n=92** |
| *SHIGELLA FLEXNERI* | 43 (65) | 23 (64) | 52 (75) | 39 (66) | 53 (60) | 39 (54) | 27 (64) | 19 (90) | 49 (80) | 60 (65) |
| *SHIGELLA SONNEI* | 3 (4) | 3 (8) | 5 (7) | 9 (15) | 11 (12) | 7 (10) | 7 (17) | 1 (5) | 5 (8) | 17(19) |
| *SHIGELLA SPECIES* | 5 (8) | 7 (19) | 6 (9) | 3 (5) | 8 (9) | 10 (14) | 5 (12) | 0 (0) | 4 (7) | 0 (0) |
| *SHIGELLA BOYDII* | 10 (15) | 1 (3) | 4 (6) | 3 (5) | 6 (6) | 7 (10) | 0 (0) | 1 (5) | 2 (3) | 12 (13) |
| *SHIGELLA DYSENTERIAE* | 5 (8) | 2 (6) | 2 (3) | 5 (9) | 11 (12) | 9 (12) | 3 (7) | 0 (0) | 1 (2) | 3 (3) |
