# Supplementary material for: Population-based incidence and antimicrobial susceptibility patterns of shigellosis among children and adults from rural and urban Kenya, 2010–2019
Source: PLoS One. 2026 Mar 26;21(3):e0330888. doi: 10.1371/journal.pone.0330888 (PMC13020798; doi:10.1371/journal.pone.0330888)
Supplement: S4 Table — (DOCX) [file pone.0330888.s005.docx]

S4 Table: Crude and adjusted annual *Shigella* incidence rates in Asembo and Kibera, Kenya, 2010-2019

|  | **Asembo** | | | **Kibera** | | |
| --- | --- | --- | --- | --- | --- | --- |
| **Year** | **Crude Incidence [95% CI]** | **Adjusted incidence1 [95% CI]** | **Adjusted Incidence2 [95% CI]** | **Crude Incidence [95% CI]** | **Adjusted incidence1 [95% CI]** | **Adjusted Incidence2 [95% CI]** |
| 2010 | 121 [25-190] | 651 [428-1013] | 1087 [707-1757] | 235 [43-402] | 467 [340-642] | 672 [492-920] |
| 2011 | 133 [21-220] | 709 [480-1135] | 1227 [826-1991] | 129 [19-212] | 356 [228-535] | 509 [326-773] |
| 2012 | 94 [23-154] | 584 [371-995] | 978 [610-1737] | 255 [48-433] | 875 [594-1509] | 1330 [923-2225] |
| 2013 | 115 [21-198] | 264 [182-364] | 514 [351-728] | 222 [45-373] | 427 [300-614] | 601 [428-855] |
| 2014 | 242 [50-422] | 488 [378-627] | 939 [720-1246] | 331 [69-509] | 551 [437-685] | 685 [544-857] |
| 2015 | 105 [19-189] | 238 [162-330] | 570 [390-816] | 298 [55-492] | 432 [330-547] | 591 [452-755] |
| 2016 | 72 [15-114] | 146 [88-216] | 275 [158-432] | 178 [35-290] | 245 [173-326] | 439 [304-610] |
| 2017 | 258 [48-391] | 386 [303-479] | 825 [636-1074] | 91 [13-138] | 127 [75-188] | 213 [122-324] |
| 2018 | 112 [25-193] | 127 [88-169] | 313 [204-460] | 263 [60-436] | 331 [249-425] | 562 [423-736] |
| 2019 | 114 [21-187] | 129 [90-172] | 278 [186-397] | 391 [76-654] | 435 [351-528] | 789 [625-995] |
| Overall | 137 [66-499] | 361 [78-1084] | 684 [151-2332] | 239 [121-866] | 433 [167-2678] | 647 [288-3887] |

*****Incidence rates were adjusted first for the proportion who were sampled among diarrhea cases, and second for proportion of cases with diarrhea at home who sought care in any clinic other than the surveillance clinic
