# Supplementary material for: Population-based incidence and antimicrobial susceptibility patterns of shigellosis among children and adults from rural and urban Kenya, 2010–2019
Source: PLoS One. 2026 Mar 26;21(3):e0330888. doi: 10.1371/journal.pone.0330888 (PMC13020798; doi:10.1371/journal.pone.0330888)
Supplement: Attachment — Submitted filename: Point_by_Point Responses_09.11.2025.docx [file pone.0330888.s007.docx]

November 09, 2025

To: *Prof. Emily Chenette*

*Editor-in-Chief, PLOS One*

1875 Mission Street

Suite 103 #188 San Francisco, CA 94103

United States

Dear Prof. *Emily*,

Please find attached our revised manuscript (#PONE-D-25-40297) entitled, “***Population-based incidence and antimicrobial susceptibility patterns of shigellosis among children and adults from rural and urban Kenya, 2010-2019***” submitted for consideration as an original article in *PLOS One.*

We are grateful to you and the reviewers for the thoughtful and constructive feedback. We have revised the manuscript in response to all comments, which we also wish to thank you since it has has improved its clarity and rigor.

A detailed, point-by-point response to each comment is included below, referencing the corresponding changes and line numbers in the revised, tracked version of the manuscript.

Thank you again for considering our resubmission. We appreciate the opportunity to improve our work and look forward to your positive evaluation.

Yours sincerely,

On behalf of the co-authors,

***Correspondence**: Richard Omore; KEMRI-CGHR, P.O Box 1578-40100, Kisumu, Kenya

E-mail: [omorerichard@gmail.com](mailto:omorerichard@gmail.com); Phone: +254-728-813-788

**Point-by-point response to Editorial and Reviewer Comments**

We sincerely appreciate the invaluable comments and suggestions provided by the editors, which we believe have contributed in improving the quality of this work. Below, we present a point-by-point response to each of the remarks, indicating the corresponding revisions made in the manuscript and referencing the relevant line numbers in the tracked version.

**Editorial Comments to Author:**

Journal requirements:

When submitting your revision, we need you to address these additional requirements.

**Comment# 1**: Please ensure that your manuscript meets PLOS ONE's style requirements, including those for file naming. The PLOS ONE style templates can be found at

<https://journals.plos.org/plosone/s/file?id=wjVg/PLOSOne_formatting_sample_main_body.pdf> and

<https://journals.plos.org/plosone/s/file?id=ba62/PLOSOne_formatting_sample_title_authors_affiliations.pdf>

**Response to comment #1:** Thank you for this comment. We have revised the manuscript to align with the journal’s style requirements. Specifically, we reformatted Level 1 and Level 2 headings, bolded figure captions and table titles, confirmed file naming convention, and removed funding and competing interests information from the Acknowledgments section as suggested.

**Comment #2:** Thank you for stating the following financial disclosure:

“This study was funded by the US Centers for Disease Control and Prevention (CDC) in Atlanta USA through a cooperative agreement with KEMRI.”

Please state what role the funders took in the study.  If the funders had no role, please state: "The funders had no role in study design, data collection and analysis, decision to publish, or preparation of the manuscript."

If this statement is not correct you must amend it as needed.

Please include this amended Role of Funder statement in your cover letter; we will change the online submission form on your behalf.

**Response to comment #2:** Thank you for this comment. We have updated the financial disclosure statement, which now reads: “*This study was funded by the US Centers for Disease Control and Prevention (CDC) in Atlanta USA through a cooperative agreement with KEMRI. The funders had no role in study design, data collection and analysis, decision to publish, or preparation of the manuscript.*”. We have included the amended funder statement in the cover letter as suggested.

**Comment #3:** Thank you for stating the following in the Funding Section of your manuscript:

“This study was funded by the US Centers for Disease Control and Prevention (CDC) in Atlanta USA through a cooperative agreement with KEMRI.”

We note that you have provided funding information that is currently declared in your Funding Statement. However, funding information should not appear in the Acknowledgments section or other areas of your manuscript. We will only publish funding information present in the Funding Statement section of the online submission form.

Please remove any funding-related text from the manuscript and let us know how you would like to update your Funding Statement. Currently, your Funding Statement reads as follows:

“This study was funded by the US Centers for Disease Control and Prevention (CDC) in Atlanta USA through a cooperative agreement with KEMRI.”

Please include your amended statements within your cover letter; we will change the online submission form on your behalf.

**Response to comment #3:** We appreciate this comment. We have now removed funding and competing interests information statements from the Acknowledgments section in the revised version as per your guidelines. We have included the amended funder statement in the cover letter.

**Comment #4:** In the online submission form you indicate that your data is not available for proprietary reasons and have provided a contact point for accessing this data. Please note that your current contact point is a co-author on this manuscript. According to our Data Policy, the contact point must not be an author on the manuscript and must be an institutional contact, ideally not an individual. Please revise your data statement to a non-author institutional point of contact, such as a data access or ethics committee, and send this to us via return email. Please also include contact information for the third-party organization, and please include the full citation of where the data can be found.

**Response to comment #4:** Thank you for this comment. We have revised the data availability statement accordingly (Lines 332-335) and now reads as follows:

“*The data used in this study are owned by the Kenya Medical Research Institute (KEMRI). Access to the data may be granted upon reasonable request to the* program ([info.pbids@kemri.go.ke](mailto:info.pbids@kemri.go.ke)) or *Head of the KEMRI Scientific and Ethics Review Unit (Email:* [*seru@kemri.go.ke*](mailto:seru@kemri.go.ke) *or* [*serukemri@gmail.com*](mailto:serukemri@gmail.com)*), subject to KEMRI’s institutional data access and sharing policies.*”

**Comment #5:** Please include captions for your Supporting Information files at the end of your manuscript, and update any in-text citations to match accordingly. Please see our Supporting Information guidelines for more information: <http://journals.plos.org/plosone/s/supporting-information>.

**Response to comment #5:** Thank you for this comment. We have now added the captions for the supporting information at the end of the revised manuscript (Lines 466-472).

**Comment #6:** If the reviewer comments include a recommendation to cite specific previously published works, please review and evaluate these publications to determine whether they are relevant and should be cited. There is no requirement to cite these works unless the editor has indicated otherwise.

**Response to comment #6:** We have not received any recommendations for citations at the moment, but we appreciate the guidance.

**Additional Editor Comments:**


**Comment #7:** To ensure your figures meet our technical requirements, please review our figure guidelines: <https://journals.plos.org/plosone/s/figures>

You may also use PLOS’s free figure tool, NAAS, to help you prepare publication quality figures: <https://journals.plos.org/plosone/s/figures#loc-tools-for-figure-preparation>.

NAAS will assess whether your figures meet our technical requirements by comparing each figure against our figure specifications.

**Response to comment #7:** Thank you for this comment. We wish to confirm that the figures were generated using NAAS and meet the journal technical requirements.
